# Looking ahead: where to next for animal models of bronchopulmonary dysplasia?

**DOI:** 10.1007/s00441-016-2534-3

**Published:** 2016-12-05

**Authors:** Claudio Nardiello, Ivana Mižíková, Rory E. Morty

**Affiliations:** 1 Department of Lung Development and Remodelling, Max Planck Institute for Heart and Lung Research, Parkstrasse 1, 61231 Bad Nauheim, Germany; 2 Department of Internal Medicine (Pulmonology), University of Giessen and Marburg Lung Center (UGMLC), Giessen, Germany

**Keywords:** Bronchopulmonary dysplasia, Animal model, Hyperoxia, Ventilation, Mouse

## Abstract

Bronchopulmonary dysplasia (BPD) is the most common complication of preterm birth, with appreciable morbidity and mortality in a neonatal intensive care setting. Much interest has been shown in the identification of pathogenic pathways that are amenable to pharmacological manipulation (1) to facilitate the development of novel therapeutic and medical management strategies and (2) to identify the basic mechanisms of late lung development, which remains poorly understood. A number of animal models have therefore been developed and continue to be refined with the aim of recapitulating pathological pulmonary hallmarks noted in lungs from neonates with BPD. These animal models rely on several injurious stimuli, such as mechanical ventilation or oxygen toxicity and infection and sterile inflammation, as applied in mice, rats, rabbits, pigs, lambs and nonhuman primates. This review addresses recent developments in modeling BPD in experimental animals and highlights important neglected areas that demand attention. Additionally, recent progress in the quantitative microscopic analysis of pathology tissue is described, together with new in vitro approaches of value for the study of normal and aberrant alveolarization. The need to examine long-term sequelae of damage to the developing neonatal lung is also considered, as is the need to move beyond the study of the lungs alone in experimental animal models of BPD.

## Introduction

The lung is the key organ of gas exchange in mammals; it undertakes the transport of oxygen from inspired air into the bloodstream and, concomitantly, the transport of carbon dioxide out of the circulating blood, which is subsequently exhaled and thus removed from the body. This gas exchange is facilitated by the highly organized structure of the alveolus, the gas-exchange unit of the lung, where the inspired air is brought into close proximity to the circulating blood (Hsia et al. 2016). The close proximity of air and blood is facilitated by the delicate alveolo-capillary barrier, a double-layered barrier consisting of alveolar epithelial cells that line the alveolar units containing the inspired air and that are intimately associated with the endothelial cells that form the capillaries of the pulmonary circulation carrying deoxygenated blood through the lung (Hsia et al. 2016). The blood-air barrier is very thin, namely 200 nm–2 μm and is permeable to many gases including oxygen and carbon dioxide, thus facilitating gas exchange. Gas exchange occurs by Fick’s Law (Fick 1855) and is a passive process that is directly determined by (1) the surface area available over which the gases can diffuse, (2) the distance across which the gas molecules must diffuse and (3) the concentration gradient. The broader objective of lung development is to generate a gas-exchange structure that satisfies these three conditions. The structure should have a large surface area and a thin diffusion barrier and should facilitate the establishment of a steep concentration gradient for gas diffusion in the correct direction. To this end, lung development is initiated with the emergence of the respiratory diverticulum, which is a ventral outgrowth of the foregut endoderm, very early during embryonic development (the fourth week of gestation in humans). Tracheo-esophageal ridges form from longitudinal mesodermal folds that “pinch off” to separate the trachea from the esophagus (Herriges and Morrisey 2014; Morrisey et al. 2013; Morrisey and Hogan 2010; Warburton et al. 2005). Lung development then proceeds via the formation of the trachea, which subsequently generates the bronchial tree, forming the conducting airways that develop largely in parallel with the vasculature that forms the pulmonary circulation (Rawlins 2011). Subsequent lung development over the canalicular, saccular and alveolar stages generates the alveolar gas-exchange units (Morty et al. 2009). To satisfy the needs of Fick’s Law, lung development strives to generate (1) a large number of small alveoli, thus producing an extremely large gas-exchange surface area and (2) as thin an alveolo-capillary barrier as possible to minimize the distance that the diffusing gases must transit. Furthermore, the proper development of the conducting airways and conducting blood vessels allows continuous blood flow in the capillaries and constant air flow (by breathing) thereby maintaining a steep concentration gradient at the site of the alveolo-capillary barrier. When lung development proceeds correctly, the lung structure that is generated is perfectly suited to provide optimal gas exchange (Herriges and Morrisey 2014; Warburton et al. 2010; West 2013).

Perturbations to lung development result in lungs that are defective for optimal gas exchange. These perturbations result in a clinically relevant disease, the most prevalent of which is bronchopulmonary dysplasia (BPD; Kinsella et al. 2006; Martin and Fanaroff 2013), a common complication of preterm birth originally described by Northway and coworkers in 1967 (Northway et al. 1967). Preterm infants often present with acute respiratory failure (neonatal or infant respiratory distress syndrome) that requires oxygen supplementation, in the most extreme cases, by mechanical ventilation. In affected infants, a combination of infection, inflammation and oxygen toxicity, together with physical forces generated by baro- and volu-trauma from mechanical ventilation, appreciably stunts the post-natal growth and maturation of the developing lung (Jain and Bancalari 2014). Over many years, because of changes in the medical management of pre-term birth, the histopathological picture of BPD has changed, moving from lung damage caused by aggressive mechanical ventilation and oxygen toxicity (“old BPD”) to a pathology that is distinctly characterized by disturbances to lung developmental pathways (“new BPD”; Day and Ryan 2016). This stunting impacts all elements of lung development and the blunted alveolar development generates fewer alveoli of a larger size; hence, the gas-exchange surface area is smaller than it should be. Additionally, lungs from affected infants are characterized by thickened alveolar walls, which increase the gas-diffusion distance between the inspired air in the alveoli and the circulating blood in the capillaries. Furthermore, a dysmorphic pulmonary vasculature develops, which may cause pulmonary hypertension, thus complicating blood flow through the lung (Baker and Abman 2015; Hadchouel et al. 2014). BPD is associated with significant morbidity and mortality in the neonatal intensive care unit and survivors exhibit long-term consequences that persist into adulthood (Baraldi and Filippone 2007; Hilgendorff and O’Reilly 2015; Wong et al. 2008). Disturbances to alveolar structure and to the pulmonary vasculature are not exclusive to BPD and can also be seen in infants with congenital diaphragmatic hernia (CDH; Sluiter et al. 2012; Veenma et al. 2012). The improvements in the medical management of preterm birth underlying the transition from “old BPD” to “new BPD” have paradoxically led to an increase in the incidence of BPD, because preterm infants born earlier and earlier now survive better (Jobe 2011, 2012; Jobe and Ikegami 1998; Madurga et al. 2013; Northway 1992); for example, preterm infants born in the 25–28 week range now have >90 % survival (Stoll et al. 2010). Thus, BPD represents an increasing clinical burden, underscoring the need for a better understanding of the aberrant lung development in affected neonates. Whereas autopsy material from BPD non-survivors presents one avenue for the exploration of pathogenic mechanisms at play in the lungs of affected patients, this material is increasingly rare and difficult to obtain, because the survival rate of BPD patients has steadily improved over time. Therefore, most studies addressing pathogenic mechanisms that drive BPD pathology are performed in experimental animals. These experimental animal models of BPD also serve as an important means of evaluating candidate therapeutic interventions, such as novel lead compounds for drug development.

## State of the art: pros and cons of animal models of BPD

Modeling BPD in experimental animals has been the subject of a large number of recent reviews; therefore, no attempt will be made here to re-review all of the available models. For this, the reader is directed to a series of state-of-the-art reviews on modeling BPD in mice (Berger and Bhandari 2014), rats (O’Reilly and Thébaud 2014), rabbits (D’Angio and Ryan 2014; Manzano et al. 2014), pre-term lambs (Albertine 2015), pre-term pigs (Arrindell et al. 2015; Caminita et al. 2015) and baboons (Yoder and Coalson 2014). Additionally, the utility of these models for the study of disease mechanisms (Hilgendorff et al. 2014) and therapy development (Jobe 2015) has also recently been reviewed.

BPD is most commonly modeled in mice and rats. The reasons for this are manifold. Mice and rats have relatively short gestation times (Table 1) and thus, studies that monitor post-natal lung maturation can be conducted relatively quickly, since lung development in both mice and rats is largely complete within the first 2 and 3 weeks of life, respectively. Additionally, several other practical advantages to working with mice and rats can be mentioned: both mice and rats are readily available and easy to maintain compared with larger vertebrates. Moreover, fewer concerns are raised by regulatory agencies against experimental studies on rats and mice, than, for example, experimental studies on non-human primates. Mice additionally offer a wide availability of many transgenic lines, although the advent of the CRISPR/Cas9 system should make the generation of transgenic animals such as rats both easier and cheaper. The smaller size of mice and rats means that smaller amounts of pharmacological agents are required compared with the same dosing of an agent in a larger animal.

**Table 1:** Stages of mouse and rat lung development compared with that of humans (*E* embryonic day, *P* postnatal day)

Stage	Gestational age		
	Mouse (days)	Rat (days)	Human (weeks)
Embryonic	E9-E11.5	E8-E13	3–7
Pseudoglandular	E11.5-E16.5	E18-E18	5–17
Canalicular	E16.5-E17.5	E18-E20	16–29
Saccular	E17.5-P5	E20-P5	24–38
Alveolar	P5-P28	P5-P30	32-adolescence

Both mice and rats are delivered at term in the saccular stage of lung development; this fact is often used to justify the superiority of mice and rats as model animals for BPD, since preterm infants that develop BPD are also delivered in the saccular stage of lung development. Thus, term mouse and rat models appear to be useful models of pre-term birth. However, we should also keep in mind that, when term mice and rats are delivered in the saccular stage, these newborn rodent pups are competent for proper gas exchange and do not require oxygen supplementation as a life-saving intervention; this is in marked contrast to preterm human neonates with or at risk for the development of BPD.

The small size of mice and rats at birth is also a disadvantage. Newborn mouse and rat pups are notoriously difficult to intubate. This is important, since mechanical ventilation is a highly clinically relevant, injurious stimulus for BPD. Thus, the maintenance of intubated ventilated newborn mouse and rat pups over prolonged periods of time is desirable in order to assess the impact on post-natal lung maturation. Tissue and cell stretch and lung distention are proposed to be important causes of tissue damage to the developing lung (Hussein et al. 2013) emphasizing the need for more ventilation-based models to study the impact of mechanical ventilation on post-natal lung maturation. For example, an 8-h aggressive mechanical ventilation period (tidal volume 15 μl/g) of 5-day-old mouse pups with room air stunts lung alveolarization, whereas a comparatively gentler mechanical ventilation protocol (tidal volume 8 μl/g) does not impact alveolarization (Ratner et al. 2013). Currently, mechanical ventilation can only be undertaken for short periods of time (up to about 24 h; Berger and Bhandari 2014; Bland et al. 2008) but not beyond. In this sense, the larger animal models are clearly superior.

The small size of newborn mouse and rat pups is also a source of concern with regard to substance administration. Parenteral administration of pharmacological agents generally must be via the intraperitoneal route, since intravenous injection in newborn mouse and rat pups is not possible. The small size and delicate skin of newborn mouse and rat pups also makes dosing frequency difficult. For substances with short half-lives, repeat dosing over short intervals is generally not possible, since repeated injection at the same injection site (one is short of options in newborn mouse pups) creates local trauma and necrosis and the placement of a mini-pump is impractical. These drawbacks again highlight the utility of the larger animal models of BPD. Scope for the improvement of the delivery of agents to newborn mouse pups does however exist. Whereas many pharmacological agents are short-lived, some chemical substances have pronounced in vivo stability. One case in point is that of antagomiRs, which are synthetic inhibitors of microRNA. MicroRNA are emerging as exciting modulators of normal and aberrant lung alveolarization (Nardiello and Morty 2016). The extraordinary stability of antagomiRs makes antagomiRs suitable as test substances for examining, for example, delivery to newborn pups via the maternal route, shortly prior to birth. Such maternal transmission studies might markedly improve agent delivery to newborn rodent pups, although such studies have yet to be reported in the literature. Along these lines, other possible routes of the administration of pharmacological and other agents include the intra-nasal route, which would be particularly relevant in the case of respiratory viruses such as respiratory syncytial virus (RSV) and influenza virus, which are known to complicate clinical BPD. Additionally, the intrauterine administration of substances such as bacterial lipopolysaccharide has been employed, to assess an impact of sterile inflammation on post-natal lung alveolarization (Ueda et al. 2006). To date, no side-by-side comparison has been published that has assessed the comparative efficacy of substance administration by using various routes of substance administration in utero, via the maternal route, or directly to newborns.

In spite of the drawbacks highlighted above, rodent models of BPD are the predominant model in widespread use today. This predominance has also led to a substantial variance in the injurious insults applied. A wide variety of mouse strains are available and many strains are employed on a mixed strain background. Whereas the C57BL/6 is the most widely-employed mouse strain, studies have also been reported involving BALB/c, FVB/n and C3H background strains and mixed backgrounds containing some 129S strain components (Silva et al. 2015). This concerns hyperoxia studies, since tremendous variation is present in the susceptibility of adult mice of different strains to hyperoxia (Hudak et al. 1993; Whitehead et al. 2006). This use of diverse mouse strains sometimes makes the comparisons of independent studies problematic. A systematic comparison of the susceptibility of the various mouse strains to hyperoxia, particularly concerning the impact of hyperoxia and mechanical ventilation on post-natal lung maturation, is a study that urgently needs to be conducted in order to allow investigators to make informed decisions about which mouse strain might be useful in studies in experimental mouse models of BPD. The importance of this idea is underscored by the tremendous influence of the mouse strain on fibrogenic responses to transforming growth factor-β (Kolb et al. 2002), which is a known mediator of aberrant lung development in the hyperoxia-based mouse model of BPD (Alejandre-Alcázar et al. 2007; Nakanishi et al. 2007).

Another area in which tremendous variation in animal models is evident is in the degree of oxygen injury employed. Term mouse and term rat models can be used, with a range of oxygen injury protocols being currently implemented. These are summarized (evaluating literature from the past 3 years) in Table 2. Although the use of diverse oxygen injury protocols is fruitful, a standardized protocol would also be helpful so that studies undertaken by diverse groups can be directly compared side-by-side. To this end, a systematic comparison of the impact of various oxygen concentrations would be of great value for the community. In this respect, a study of the impact of gradual versus abrupt changes in inspired oxygen concentrations on lung alveolarization needs to be conducted in order to consider the translational implications of gradual weaning. Along these lines, from an animal welfare point of view, the determination of the “windows” of post-natal lung development that are sensitive to oxygen injury would be advantageous. Currently, many investigators simply expose newborn rodents to elevated oxygen concentrations for fixed periods of time, for example, from the day of birth to post-natal day (P)14, at which time point the bulk alveolarization that takes place over the immediate postnatal period is largely complete. However, much shorter durations of hyperoxia exposure (such as a window spanning the “peak” period of secondary septation in mice; say P3-P7) would be sufficient to recapitulate the lung histopathological characteristics of BPD. Such a systematic study examining the effects of a defined oxygen concentration over discrete windows of postnatal lung development in rodents has yet to be performed. Thus, whereas the use of mouse and rat models is widespread, these models are not without substantial drawbacks leading some investigators to explore other experimental animal models of preterm birth and BPD.

**Table 2:** Variance in oxygen-exposure protocols in rodent models of bronchopulmonary dysplasia (*LPS* lipopolysaccharide, *P* postnatal day)

Oxygen level (%)	Oxygen duration	Protocol notes	Reference
40	P1-P7	Recovery in 21% O_2_ P7-P21	Wang et al. 2014
50/10	P1-P14	Oscillation; recovery in 21% O_2_ P14-P21	M. Chang et al. 2013
60	P1-P14		Ahlfeld et al. 2013; Belcastro et al. 2015; Masood et al. 2014
70	P3-P13		Bachiller et al. 2013
75	P1-P7		Harijith et al. 2013
75	P1-P14		Monz et al. 2013
75	P1-P14	Prenatal hypoxia (10% O_2_)	Gortner et al. 2013
75	P2-P17		Popova et al. 2014
75	P3-P27		Anyanwu et al. 2014
80	P6-P8		Weichelt et al. 2013
80	P1-P7	Prenatal LPS, recovery in 21% O_2_ P7-P14	Lee et al. 2014
80	P1-P15		Dayanim et al. 2014
80	P1-P7	Recovery in 21% O_2_ P8-P21	Pham et al. 2014
80	P1-P28	Recovery in 21% O_2_ P28-P56	Tibboel et al. 2015
85	P1-P7		James et al. 2013; Koskinen et al. 2014; Witsch et al. 2014a, 2014b
85	P2-P9		Park et al. 2013
85	P1-P10		Madurga et al. 2014
85	P1-P14		Ahlfeld et al. 2013; Britt et al. 2013; James et al. 2013; Raffay et al. 2013
85	P4-P14		Alphonse et al. 2014
85	P2-P8	Recovery in 21% O_2_ P8-P16	Park et al. 2013
85	P1-P14	Recovery in 21% O_2_ P14-P28	James et al. 2013; Raffay et al. 2013; Rieger-Fackeldey et al. 2014
85/gradient	P1-P28	85% O_2_ P1-P14, followed by gradient from 85% O_2_ to 21% O_2_ between P14 and P28	Rieger-Fackeldey et al. 2014
90	P3-P10	Recovery in 21% O_2_ P10-P14	Ramachandran et al. 2015
90	P1-P14		Ahn et al. 2015
90	P2-P15		Hummler et al. 2013
90	P1-P14	Recovery in 21% O_2_ P14-P15	Alapati et al. 2014
90	P1-P15		Sutsko et al. 2013
90	P2-P15	Recovery in 21% O_2_ P15-P28	Miranda et al. 2013
90	P1-P15	Recovery in 21% O_2_ P15-P29	Sutsko et al. 2013
95	P1-P7	Initiated pre-natally	Richter et al. 2014
90/60	P1-P21	90% O_2_ P1-P14, 60% O_2_ P14-P21	Y.S. Chang et al. 2013
95	P4-P14		Alphonse et al. 2014
95	P1-P14		Alphonse et al. 2014; McKenna et al. 2014; Vadivel et al. 2014
95	P1-P6	Recovery in 21% O_2_ P6-P21	Sakurai et al. 2013
95	P1-P7	Recovery in 21% O_2_ P7-P56	Ahlfeld et al. 2015
100	P1-P3.5		Kawamura et al. 2013
100	P1-P7		Sureshbabu et al. 2015
100	P1-P7	With LPS application	Syed and Bhandari 2013
100	P1-P10		Bhattacharya et al. 2014; Martin et al. 2014; Wagenaar et al. 2013a, 2013b
100	P1-P4	Recovery in 21% O_2_ P4-P56	Buczynski et al. 2013

Rabbits represent the middle-ground between small vertebrates such as rats and mice and larger vertebrates such as sheep, pigs and nonhuman primates (Table 3). Unlike mice and rats, rabbit kits can be delivered preterm by caesarian section and can then be subjected to mechanical ventilation (as instrumentation is substantially easier in rabbit kits than in newborn mouse and rat pups). A preterm rabbit model with hyperoxia exposure has been described (Manzano et al. 2014; Richter et al. 2014; Salaets et al. 2015) and the use of preterm rabbits continues to be refined (D’Angio and Ryan 2014; Manzano et al. 2014).

**Table 3:** Stages of lung development in larger animals compared with that of humans

Stage	Gestational age (days)				
	Rabbit	Sheep	Pig	Baboon	Human
Term	32	147	115	168–185	280
Embryonic	Up to 18	Up to 40	Up to 25	Up to 42	Up to 42
Pseudoglandular	21–24	40–80	22–56	Up to 80	52–112
Canalicular	24–27	80–110	56–98	80–120	112–168
Saccular	From 27	110–130	From 99	120–140	From 168
Alveolar	From 30	From 130	From 104	From 140	From 252

Prior to the development of the rabbit model, preterm lambs proved most useful for the modeling of BPD and continue to be used today (Albertine 2015; Brew et al. 2013; Collins et al. 2013a; Galinsky et al. 2013; Hillman et al. 2013; Kallapur et al. 2013; Null et al. 2014). The ability to deliver lambs preterm is particularly appealing for translationally relevant modeling of BPD and the larger size of preterm lambs, compared with preterm rabbits, makes instrumentation and mechanical ventilation easier. Clearly, substantial costs are associated with the preterm lamb model. Apart from lambs, renewed interest has been shown in the use of term and preterm piglets to study BPD (Bartlett et al. 2013; Caminita et al. 2015); preterm piglets exhibit ventilation inadequacies, together with clinically comparable risks for the development of respiratory disease syndrome. Thus, piglets represent an alternative to nonhuman primates and lambs.

Primates have been considered the most translationally relevant models for BPD; however, the extraordinary costs of primate studies, together with complex ethical considerations, have led to a decline in the use of primates to model BPD. Two widely used preterm primate models are available (Yoder and Coalson 2014): (1) the 140-day premature baboon model in which preterm baboons are ventilated with high pressures and an FiO_2_ of up to 100 % oxygen for several days is believed histopathologically to mimic “old BPD” (Escobedo et al. 1982); (2) the 125-day premature baboon model in which preterm baboons are ventilated with low tidal volume or nasal continuous positive airway pressure and an FiO_2_ tailored to individual need (up to and beyond 28 days) is believed to mimic more closely “new BPD” (Coalson et al. 1999; Maniscalco et al. 2002). The latter model is occasionally combined with intrauterine exposure to *Ureaplasma*, which frequently results in preterm labor (Yoder and Coalson 2014). The relative phylogenetic closeness of non-human primates to humans, compared with rodents and the ability to deliver non-human primates pre-term have clear advantages for translational research into new BPD. Primates are also often susceptible to the same or related viruses that complicate clinical BPD. For example, newborn baboons can be infected with RSV and also develop pathological features consistent with RSV infection in human neonates (Papin et al. 2013). This is important, since RSV remains an important clinical problem in a neonatal intensive care setting (Carpenter and Stenmark 2004). In addition to clinical and anatomic parallels, non-human primates may be more useful for the study of the long-term sequelae of BPD associated with pre-term birth, including extra-pulmonary sequelae such as neurodevelopmental outcomes, since cognition studies on baboons are well established (Zürcher et al. 2010). From a translational medicine point of view (for example, the assessment of novel therapeutic approaches or the assessment of long-term neurodevelopmental outcomes), the baboon models are unquestionably an ideal model, despite grave ethical concerns and considerable cost. In contrast, for the study of pathogenic pathways, whereby artificial modulation of gene expression is required implicate causality, rodent models remain the method of choice in terms of cost, convenience and tool availability.

In summary, a spectrum of experimental animals has proved useful as model organisms to study BPD, with particular advantages and disadvantages associated with the use of the various model organisms. Given issues of cost, ethics, time and the availability of transgenic animals, the mouse will probably remain the most widely used model for BPD. Whereas hyperoxia and mechanical ventilation have been highlighted as the predominant injurious stimuli for BPD models, much scope remains for an improvement of the way that we induce arrested lung development in BPD models. In this respect, much interest has been shown in sterile and infection-driven inflammation, such as intrauterine inflammation, either singly or in combination with mechanical ventilation or hyperoxia exposure, as a “second hit” (Collins et al. 2013a, 2013b; Yoo et al. 2013). These inflammation-based models are important and clinically relevant BPD models that demand more development and increased use.

## Assessment of lung structure: stereological and three-dimensional approaches

Most BPD models today are employed in studies involving perturbations to the development of lung structure. Of note at this junction, the clinical definition of BPD does not in any way include structural elements of lung development. In a neonatal intensive care setting, the diagnosis of BPD is currently based on the need for supplemental oxygen for at least 28 days after birth and its severity is graded according to the respiratory support required at 36 postmenstrual weeks (Jobe 2011, 2012). This does not directly take into account lung structure or perturbations to lung development. Nevertheless, most experimental investigations address the mechanisms underlying stunted lung development and exploit animal models to explore candidate interventions with the aim of promoting lung growth and alveolarization. Thus, the ability effectively to quantify changes in lung structure, most importantly with regard to the size and abundance of the alveoli, the gas-exchange surface area, the vascularization of the lung and the thickness of the septal walls, is clearly important.

The quantification of lung structure is currently undergoing a revolution. Historically, structural analysis of the lung has been undertaken in paraffin-embedded lung tissue and lung structure was analyzed through the determination of the mean linear intercept (MLI) and radial alveolar count (RAC) as surrogates of the size of the alveoli (Silva et al. 2015). These determinations are generally made by direct measurements of the distance between adjacent walls of an alveolus by means of a slide rule. The thickness of the alveolar wall can be similarly measured by visual inspection; however, both approaches are not unbiased. Of greater concern is the distortion of the lung structure that occurs during the tissue processing, namely, the dehydration and rehydration of the lung for paraffin embedding (Schneider and Ochs 2014). These are important concerns, although we must also acknowledge that these more classical approaches form the basis of most of the important observations made to date regarding not only pathological mechanisms of BPD but also drug discovery (Liao et al. 2015; Olave et al. 2015; Schneider and Ochs 2014; Tibboel et al. 2013). To address concerns about bias in analyses and the generation of artifacts during tissue processing, much effort has been expended on replacing paraffin embedding with embedding in plastic resins, together with the treatment of lung tissue with osmium and uranium in order to preserve lung structure (Schneider and Ochs 2014). Additionally, design-based stereology methodologies are unbiased, have higher precision (Schneider and Ochs 2013; Tschanz et al. 2014a), and thus represent an advance over the classical MLI and RAC methods (Mühlfeld and Ochs 2013; Ochs and Mühlfeld 2013). Design-based stereology has recently been applied to the analysis of lung structure in newborn mice (Madurga et al. 2014, 2015; Mižíková et al. 2015) and rats (Tschanz et al. 2014b) and these technologies continue to evolve. Whereas the estimation of alveoli number, gas-exchange surface area and the thickness of the septal wall has developed well, the quantification of vascular structure remains under-developed. Historically, surrogate measures for capillary “density” have been the quantification of immune-histochemical staining of endothelial cell markers in low-power microscopic fields. However, this approach is fraught with pitfalls. Recent work addressing the quantification of capillaries in the lung by using a stereological approach is an important step in the right direction (Knust et al. 2009; Mühlfeld 2014; Willführ et al. 2015) and will no doubt continue to be refined.

If we remain with the topic of microscopy, although not in a quantitative sense, recent exciting studies concerning the three-dimensional reconstruction of serial sections of lungs from patients with BPD have revealed the presence of intrapulmonary anastomoses that would not have been detected in two-dimensional microscopy studies (Galambos et al. 2013). This highlights the tremendous utility of the three-dimensional visualization of lung structures in aberrantly developing lungs. One exciting recent report addresses the three-dimensional study of alveologenesis in mice undergoing normal lung development (Branchfield et al. 2016). These types of approaches have not been employed in lung tissue taken from animal models of BPD but this remains on the “to do list”. Furthermore, the availability of transgenic driver mice with facilitated fluorescent labeling of discrete cell types in the lung should allow for studies of cell-cell interactions during lung development. Such an experimental set-up might be used to explore endothelial-epithelial contact and whether these contacts are preserved under conditions of stunted lung growth and blunted alveolarization.

## In vitro studies

The development of the three-dimensional architecture of the alveolus demands that alveolarization is studied in intact living animals. However, elements of alveolarization can also be studied in ex vivo tissue from animal models of BPD. An increasing number of reports are appearing in the literature detailing such ex vivo studies. These approaches include the use of co-cultures (Greer et al. 2014; Lewis et al. 2015; Pieretti et al. 2014) to address cell-cell interactions and cell phenotypic transformation during alveolarization, plus in-vitro-generated lung organoids that appear to undergo processes akin to alveolarization (Dye et al. 2015; Quantius et al. 2016). Evidence has also been presented that lung sections harvested from developing mouse lungs and maintained in culture continue to undergo secondary septation (Pieretti et al. 2014). This provides a potentially exciting means of intervening in developmental and pathogenic pathways; such intervention cannot be performed in living animals for reasons of toxicity. These cultured lung explants can be manipulated, for example, through artificial stretch (Davidovich et al. 2013), which might be used to mimic the impact of mechanical ventilation in vitro. The wide availability of cell-type-specific mouse driver lines has introduced the possibility of directly assessing communication between specific cell types during alveolarization, for example, in the in vitro alveolarization studies mentioned above and also by using the previously described organoid systems in which cells derived from particular driver lines can be recombined in vitro. This might be particularly relevant, for example, to the study of the various fibroblast lineages, such as platelet-derived growth factor receptor (PDGF)-α-labeled fibroblasts, including the PDGFRα^hi^ and PDGFRα^low^ subpopulations of lung fibroblasts that have been accredited with different signaling roles in vivo during lung alveologenesis (McGowan and McCoy 2015; Ntokou et al. 2015; Ruiz-Camp and Morty 2015).

## Perspective: where do we go from here?

The modeling of BPD in animals can be improved and developed in many areas. Use of oxygen as an injurious stimulus is likely to remain a key driver of pathology in experimental animal models of BPD, irrespective of whether low levels (40 % O_2_) or high levels (85 % O_2_) of oxygen are employed. Much scope remains for the improvement of the pure hyperoxia-based models. A very large range of oxygen concentrations have been employed, from 40 % O_2_ to 100 % O_2_, in a variety of exposure protocols, with and without a “second hit”, which has both advantages and disadvantages. Amongst the advantages is that much has been learnt about how (much) oxygen injures the developing lung. However, the use of a broad range of oxygen exposure protocols has also yielded data (and conclusions) from various investigators in the field but these data are difficult to compare side-by-side. A systematic comparison of hyperoxia exposure protocols would be a most useful addition to the body of literature on animal models of BPD, as would a comparison of the effects of hyperoxia on different mouse strain backgrounds. The further development of translationally relevant “two-hit” models remains a priority area in experimental animal studies on BPD, as does the technological development of mechanical ventilation and instrumentation of very small newborn rodents.

As highlighted above, the clinical definition of BPD relates to a need for – and the degree of – oxygen supplementation at defined time-points during postnatal life. Lung structure is not considered within that definition. The lack of physiological studies on respiratory function in animal models of BPD is remarkable and warrants attention. Some investigators have taken steps to address the relationship of lung structure to the functional impairment of respiratory function (Ahlfeld et al. 2015) and more studies along these lines are urgently needed. The delineation of the degree of the stunting to alveolarization that results in an appreciable impact on gas-exchange physiology in experimental animal models of BPD would be welcome.

The rapid pace of technological development of in vivo imaging, microscopy and computational analysis highlights the utility of these approaches to the study of aberrant lung development in experimental animal models. Notable amongst these is the three-dimensional reconstruction of pathology samples, as this method might reveal anatomical disturbances not evident from two-dimensional analyses. The ability to manipulate lung sections ex vivo in cell culture brings with it new possibilities for the analysis and intervention in lung development pathways; such investigations might not be possible in intact living animals.

As a final point, we also need to broaden our horizons, both beyond the immediate post-natal period and beyond the lung. BPD is known to have sequelae that persist into adulthood and yet, our understanding of the long-term consequences of exposure to hyperoxia, pulmonary infection and inflammation and mechanical ventilation in neonates remains relatively understudied. This is a matter of intensive investigation by some groups, such as that of Michael O’Reilly at the University of Rochester and further exploration of the way that an insult to the developing lung in the postnatal period impacts lung (and general) health in later life is an important area for future work involving the use of experimental animal models of BPD. If we move beyond the lung, neonates with BPD are known to be at risk of serious extra-pulmonary morbidities, including (but not limited to) adverse neurodevelopmental outcomes and retinopathy of prematurity. Important work to address extra-pulmonary morbidity in a hyperoxia-based model of BPD in rodents has now started to emerge (Bravo-Nuevo et al. 2016; Poon et al. 2016). These issues have hardly been studied at all in experimental animal models of BPD and would most likely yield some exciting experimental data.

## Summary: neglected areas and urgent needs

The application of animal models for the study of BPD continues to be refined and improved. Several neglected areas currently warrant attention by investigators. (1) Whereas the diversity of animal models of BPD represents a strength of the field, the lack of standardization of the rodent models, in particular, the hyperoxia-based rodent models, can be problematic. Ideally, optimization studies would identify as low an FiO_2_ as possible (which would be in agreement with clinical practice and would embrace concerns about animal welfare) that still recapitulates the pathological hallmarks of BPD. Studies that systematically compare, side-by-side, the impact of diverse oxygen exposure protocols on lung alveolarization are needed. In this respect, the long-term consequences of perturbed lung development in the immediate post-natal period will be an important aspect to address in animal models. (2) The analysis of lung structure has rapidly developed over the past few years, with increased implementation of design-based stereological approaches for the analysis of selected elements of lung structure, for example, the alveoli. However, the stereological analysis of lung vascular development, in particular, the capillaries, remains under-developed. Given that perturbed vascular development is a complication of both clinical and experimental BPD, further refinement of the analysis of lung vascular structures is a priority area. With regard to imaging, the development of alternative approaches to the quantification of lung structure would be desirable. Amongst these are refinement in the visualization and analysis of lung structure in three dimensions and radiological approaches to facilitate serial measurement of the development of the lung in the same animal over an experimental time-course. (3) Although many studies addressing the pathogenesis and management of BPD in animal models utilize a structural analysis of the lung as a primary readout, few studies support these structural analyses with physiological readouts of gas exchange and lung function. Lung function analyses are routinely performed in studies on adult experimental animals, for example, by utilizing the FlexiVent system or whole-body plethysmography; however, these approaches have not been adapted to newborn or juvenile (developing) animals, largely because of technical limitations. The inclusion of lung function data is highly desirable in future studies utilizing animal models of BPD. (4) Given that clinical BPD has a massive impact on several extra-pulmonary issues, including retinopathy and neurodevelopmental outcomes, we urgently need to consider extra-pulmonary effects in animal models of BPD. Thus, much exciting work remains to be done!

## References

[CR1] Ahlfeld SK, Gao Y, Wang J, Horgusluoglu E, Bolanis E, Clapp DW, Conway SJ (2013). Periostin downregulation is an early marker of inhibited neonatal murine lung alveolar septation. Birth Defects Res A Clin Mol Teratol.

[CR2] Ahlfeld SK, Gao Y, Conway SJ, Tepper RS (2015). Relationship of structural to functional impairment during alveolar-capillary membrane development. Am J Pathol.

[CR3] Ahn SY, Chang YS, Sung DK, Yoo HS, Sung SI, Choi SJ, Park WS (2015). Cell type-dependent variation in paracrine potency determines therapeutic efficacy against neonatal hyperoxic lung injury. Cytotherapy.

[CR4] Alapati D, Rong M, Chen S, Hehre D, Hummler SC, Wu S (2014). Inhibition of beta-catenin signaling improves alveolarization and reduces pulmonary hypertension in experimental bronchopulmonary dysplasia. Am J Respir Cell Mol Biol.

[CR5] Albertine KH (2015). Utility of large-animal models of BPD: chronically ventilated preterm lambs. Am J Physiol Lung Cell Mol Physiol.

[CR6] Alejandre-Alcázar MA, Kwapiszewska G, Reiss I, Amarie OV, Marsh LM, Sevilla-Pérez J, Wygrecka M, Eul B, Köbrich S, Hesse M, Schermuly RT, Seeger W, Eickelberg O, Morty RE (2007). Hyperoxia modulates TGF-beta/BMP signaling in a mouse model of bronchopulmonary dysplasia. Am J Physiol Lung Cell Mol Physiol.

[CR7] Alphonse RS, Vadivel A, Fung M, Shelley WC, Critser PJ, Ionescu L, O’Reilly M, Ohls RK, McConaghy S, Eaton F, Zhong S, Yoder M, Thebaud B (2014). Existence, functional impairment, and lung repair potential of endothelial colony-forming cells in oxygen-induced arrested alveolar growth. Circulation.

[CR8] Anyanwu AC, Bentley JK, Popova AP, Malas O, Alghanem H, Goldsmith AM, Hershenson MB, Pinsky DJ (2014). Suppression of inflammatory cell trafficking and alveolar simplification by the heme oxygenase-1 product carbon monoxide. Am J Physiol Lung Cell Mol Physiol.

[CR9] Arrindell EL, Krishnan R, van der Merwe M, Caminita F, Howard SC, Zhang J, Buddington RK (2015). Lung volume recruitment in a preterm pig model of lung immaturity. Am J Physiol Lung Cell Mol Physiol.

[CR10] Bachiller PR, Cornog KH, Kato R, Buys ES, Roberts JD (2013). Soluble guanylate cyclase modulates alveolarization in the newborn lung. Am J Physiol Lung Cell Mol Physiol.

[CR11] Baker CD, Abman SH (2015). Impaired pulmonary vascular development in bronchopulmonary dysplasia. Neonatology.

[CR12] Baraldi E, Filippone M (2007). Chronic lung disease after premature birth. N Engl J Med.

[CR13] Bartlett JA, Albertolle ME, Wohlford-Lenane C, Pezzulo AA, Zabner J, Niles RK, Fisher SJ, McCray PB, Williams KE (2013). Protein composition of bronchoalveolar lavage fluid and airway surface liquid from newborn pigs. Am J Physiol Lung Cell Mol Physiol.

[CR14] Belcastro R, Lopez L, Li J, Masood A, Tanswell AK (2015). Chronic lung injury in the neonatal rat: up-regulation of TGFbeta1 and nitration of IGF-R1 by peroxynitrite as likely contributors to impaired alveologenesis. Free Radic Biol Med.

[CR15] Berger J, Bhandari V (2014). Animal models of bronchopulmonary dysplasia. The term mouse models. Am J Physiol Lung Cell Mol Physiol.

[CR16] Bhattacharya S, Zhou Z, Yee M, Chu CY, Lopez AM, Lunger VA, Solleti SK, Resseguie E, Buczynski B, Mariani TJ, O’Reilly MA (2014). The genome-wide transcriptional response to neonatal hyperoxia identifies Ahr as a key regulator. Am J Physiol Lung Cell Mol Physiol.

[CR17] Bland RD, Ertsey R, Mokres LM, Xu L, Jacobson BE, Jiang S, Alvira CM, Rabinovitch M, Shinwell ES, Dixit A (2008). Mechanical ventilation uncouples synthesis and assembly of elastin and increases apoptosis in lungs of newborn mice. Prelude to defective alveolar septation during lung development?. Am J Physiol Lung Cell Mol Physiol.

[CR18] Branchfield K, Li R, Lungova V, Verheyden JM, McCulley D, Sun X (2016). A three-dimensional study of alveologenesis in mouse lung. Dev Biol.

[CR19] Bravo-Nuevo A, Brandli AA, Gerhart J, Nichols J, Pitts M, Sutera CK, Assali S, Scheinfeld V, Prendergast GC, Stone J, George-Weinstein M (2016). Neuroprotective effect of Myo/Nog cells in the stressed retina. Exp Eye Res.

[CR20] Brew N, Hooper SB, Zahra V, Wallace M, Harding R (2013). Mechanical ventilation injury and repair in extremely and very preterm lungs. PLoS One.

[CR21] Britt RD, Velten M, Tipple TE, Nelin LD, Rogers LK (2013). Cyclooxygenase-2 in newborn hyperoxic lung injury. Free Radic Biol Med.

[CR22] Buczynski BW, Yee M, Martin KC, Lawrence BP, O’Reilly MA (2013). Neonatal hyperoxia alters the host response to influenza A virus infection in adult mice through multiple pathways. Am J Physiol Lung Cell Mol Physiol.

[CR23] Caminita F, van der Merwe M, Hance B, Krishnan R, Miller S, Buddington K, Buddington RK (2015). A preterm pig model of lung immaturity and spontaneous infant respiratory distress syndrome. Am J Physiol Lung Cell Mol Physiol.

[CR24] Carpenter TC, Stenmark KR (2004). Predisposition of infants with chronic lung disease to respiratory syncytial virus-induced respiratory failure: a vascular hypothesis. Pediatr Infect Dis J.

[CR25] Chang M, Bany-Mohammed F, Kenney MC, Beharry KD (2013). Effects of a superoxide dismutase mimetic on biomarkers of lung angiogenesis and alveolarization during hyperoxia with intermittent hypoxia. Am J Transl Res.

[CR26] Chang YS, Choi SJ, Ahn SY, Sung DK, Sung SI, Yoo HS, Oh WI, Park WS (2013). Timing of umbilical cord blood derived mesenchymal stem cells transplantation determines therapeutic efficacy in the neonatal hyperoxic lung injury. PLoS One.

[CR27] Coalson JJ, Winter VT, Siler-Khodr T, Yoder BA (1999). Neonatal chronic lung disease in extremely immature baboons. Am J Respir Crit Care Med.

[CR28] Collins JJ, Kallapur SG, Knox CL, Kemp MW, Kuypers E, Zimmermann LJ, Newnham JP, Jobe AH, Kramer BW (2013). Repeated intrauterine exposures to inflammatory stimuli attenuated transforming growth factor-beta signaling in the ovine fetal lung. Neonatology.

[CR29] Collins JJ, Kunzmann S, Kuypers E, Kemp MW, Speer CP, Newnham JP, Kallapur SG, Jobe AH, Kramer BW (2013). Antenatal glucocorticoids counteract LPS changes in TGF-beta pathway and caveolin-1 in ovine fetal lung. Am J Physiol Lung Cell Mol Physiol.

[CR30] D’Angio CT, Ryan RM (2014). Animal models of bronchopulmonary dysplasia. The preterm and term rabbit models. Am J Physiol Lung Cell Mol Physiol.

[CR31] Davidovich N, Huang J, Margulies SS (2013). Reproducible uniform equibiaxial stretch of precision-cut lung slices. Am J Physiol Lung Cell Mol Physiol.

[CR32] Day CL, Ryan RM (2016) Bronchopulmonary dysplasia: new becomes old again! Pediatr Res (in press) 10.1038/pr.2016.201 27682969

[CR33] Dayanim S, Lopez B, Maisonet TM, Grewal S, Londhe VA (2014). Caffeine induces alveolar apoptosis in the hyperoxia-exposed developing mouse lung. Pediatr Res.

[CR34] Dye BR, Hill DR, Ferguson MA, Tsai YH, Nagy MS, Dyal R, Wells JM, Mayhew CN, Nattiv R, Klein OD, White ES, Deutsch GH, Spence JR (2015). In vitro generation of human pluripotent stem cell derived lung organoids. eLife.

[CR35] Escobedo MB, Hilliard JL, Smith F, Meredith K, Walsh W, Johnson D, Coalson JJ, Kuehl TJ, Null DM, Robotham JL (1982). A baboon model of bronchopulmonary dysplasia. I. Clinical features. Exp Mol Pathol.

[CR36] Fick A (1855). Über Diffusion. Pogg Ann Physik Chemie.

[CR37] Galambos C, Sims-Lucas S, Abman SH (2013). Histologic evidence of intrapulmonary anastomoses by three-dimensional reconstruction in severe bronchopulmonary dysplasia. Ann Am Thorac Soc.

[CR38] Galinsky R, Polglase GR, Hooper SB, Black MJ, Moss TJ (2013). The consequences of chorioamnionitis: preterm birth and effects on development. J Pregnancy.

[CR39] Gortner L, Monz D, Mildau C, Shen J, Kasoha M, Laschke MW, Roolfs T, Schmiedl A, Meier C, Tutdibi E (2013). Bronchopulmonary dysplasia in a double-hit mouse model induced by intrauterine hypoxia and postnatal hyperoxia: closer to clinical features?. Ann Anat.

[CR40] Greer RM, Miller JD, Okoh VO, Halloran BA, Prince LS (2014). Epithelial-mesenchymal co-culture model for studying alveolar morphogenesis. Organogenesis.

[CR41] Hadchouel A, Franco-Montoya ML, Delacourt C (2014). Altered lung development in bronchopulmonary dysplasia. Birth Defects Res A Clin Mol Teratol.

[CR42] Harijith A, Pendyala S, Reddy NM, Bai T, Usatyuk PV, Berdyshev E, Gorshkova I, Huang LS, Mohan V, Garzon S, Kanteti P, Reddy SP, Raj JU, Natarajan V (2013). Sphingosine kinase 1 deficiency confers protection against hyperoxia-induced bronchopulmonary dysplasia in a murine model: role of S1P signaling and Nox proteins. Am J Pathol.

[CR43] Herriges M, Morrisey EE (2014). Lung development: orchestrating the generation and regeneration of a complex organ. Development.

[CR44] Hilgendorff A, O’Reilly MA (2015). Bronchopulmonary dysplasia early changes leading to long-term consequences. Front Med (Lausanne).

[CR45] Hilgendorff A, Reiss I, Ehrhardt H, Eickelberg O, Alvira CM (2014). Chronic lung disease in the preterm infant. Lessons learned from animal models. Am J Respir Cell Mol Biol.

[CR46] Hillman NH, Kemp MW, Noble PB, Kallapur SG, Jobe AH (2013). Sustained inflation at birth did not protect preterm fetal sheep from lung injury. Am J Physiol Lung Cell Mol Physiol.

[CR47] Hsia CC, Hyde DM, Weibel ER (2016). Lung structure and the intrinsic challenges of gas exchange. Comp Physiol.

[CR48] Hudak BB, Zhang LY, Kleeberger SR (1993). Inter-strain variation in susceptibility to hyperoxic injury of murine airways. Pharmacogenetics.

[CR49] Hummler SC, Rong M, Chen S, Hehre D, Alapati D, Wu S (2013). Targeting glycogen synthase kinase-3beta to prevent hyperoxia-induced lung injury in neonatal rats. Am J Respir Cell Mol Biol.

[CR50] Hussein O, Walters B, Stroetz R, Valencia P, McCall D, Hubmayr RD (2013). Biophysical determinants of alveolar epithelial plasma membrane wounding associated with mechanical ventilation. Am J Physiol Lung Cell Mol Physiol.

[CR51] Jain D, Bancalari E (2014). Bronchopulmonary dysplasia: clinical perspective. Birth Defects Res A Clin Mol Teratol.

[CR52] James ML, Ross AC, Nicola T, Steele C, Ambalavanan N (2013). VARA attenuates hyperoxia-induced impaired alveolar development and lung function in newborn mice. Am J Physiol Lung Cell Mol Physiol.

[CR53] Jobe AH (2011). The new bronchopulmonary dysplasia. Curr Opin Pediatr.

[CR54] Jobe AH (2012). What is BPD in 2012 and what will BPD become?. Early Hum Dev.

[CR55] Jobe AH (2015). Animal models, learning lessons to prevent and treat neonatal chronic lung disease. Front Med (Lausanne).

[CR56] Jobe AH, Ikegami M (1998). Mechanisms initiating lung injury in the preterm. Early Hum Dev.

[CR57] Kallapur SG, Kramer BW, Jobe AH (2013). Ureaplasma and BPD. Semin Perinatol.

[CR58] Kawamura T, Wakabayashi N, Shigemura N, Huang CS, Masutani K, Tanaka Y, Noda K, Peng X, Takahashi T, Billiar TR, Okumura M, Toyoda Y, Kensler TW, Nakao A (2013). Hydrogen gas reduces hyperoxic lung injury via the Nrf2 pathway in vivo. Am J Physiol Lung Cell Mol Physiol.

[CR59] Kinsella JP, Greenough A, Abman SH (2006). Bronchopulmonary dysplasia. Lancet.

[CR60] Knust J, Ochs M, Gundersen HJ, Nyengaard JR (2009). Stereological estimates of alveolar number and size and capillary length and surface area in mice lungs. Anat Rec (Hoboken).

[CR61] Kolb M, Bonniaud P, Galt T, Sime PJ, Kelly MM, Margetts PJ, Gauldie J (2002). Differences in the fibrogenic response after transfer of active transforming growth factor-beta1 gene to lungs of “fibrosis-prone” and “fibrosis-resistant” mouse strains. Am J Respir Cell Mol Biol.

[CR62] Koskinen A, Lukkarinen H, Laine J, Ahotupa M, Kaapa P, Soukka H (2014). Delay in rat lung alveolarization after the combined exposure of maternal hyperglycemia and postnatal hyperoxia. Pediatr Pulmonol.

[CR63] Lee HJ, Lee YJ, Choi CW, Lee JA, Kim EK, Kim HS, Kim BI, Choi JH (2014). Rosiglitazone, a peroxisome proliferator-activated receptor-gamma agonist, restores alveolar and pulmonary vascular development in a rat model of bronchopulmonary dysplasia. Yonsei Med J.

[CR64] Lewis KJ, Tibbitt MW, Zhao Y, Branchfield K, Sun X, Balasubramaniam V, Anseth KS (2015). In vitro model alveoli from photodegradable microsphere templates. Biomater Sci.

[CR65] Liao J, Kapadia VS, Brown LS, Cheong N, Longoria C, Mija D, Ramgopal M, Mirpuri J, McCurnin DC, Savani RC (2015). The NLRP3 inflammasome is critically involved in the development of bronchopulmonary dysplasia. Nat Commun.

[CR66] Madurga A, Mižíková I, Ruiz-Camp J, Morty RE (2013). Recent advances in late lung development and the pathogenesis of bronchopulmonary dysplasia. Am J Physiol Lung Cell Mol Physiol.

[CR67] Madurga A, Mižíková I, Ruiz-Camp J, Vadász I, Herold S, Mayer K, Fehrenbach H, Seeger W, Morty RE (2014). Systemic hydrogen sulfide administration partially restores normal alveolarization in an experimental animal model of bronchopulmonary dysplasia. Am J Physiol Lung Cell Mol Physiol.

[CR68] Madurga A, Golec A, Pozarska A, Ishii I, Mižíková I, Nardiello C, Vadasz I, Herold S, Mayer K, Reichenberger F, Fehrenbach H, Seeger W, Morty RE (2015). The H_2_S-generating enzymes cystathionine beta-synthase and cystathionine gamma-lyase play a role in vascular development during normal lung alveolarization. Am J Physiol Lung Cell Mol Physiol.

[CR69] Maniscalco WM, Watkins RH, Pryhuber GS, Bhatt A, Shea C, Huyck H (2002). Angiogenic factors and alveolar vasculature: development and alterations by injury in very premature baboons. Am J Physiol Lung Cell Mol Physiol.

[CR70] Manzano RM, Mascaretti RS, Carrer V, Haddad LB, Fernandes AR, Reyes AM, Rebello CM (2014). A hyperoxic lung injury model in premature rabbits: the influence of different gestational ages and oxygen concentrations. PLoS One.

[CR71] Martin CR, Zaman MM, Gilkey C, Salguero MV, Hasturk H, Kantarci A, Van Dyke TE, Freedman SD (2014). Resolvin D1 and lipoxin A4 improve alveolarization and normalize septal wall thickness in a neonatal murine model of hyperoxia-induced lung injury. PLoS One.

[CR72] Martin RJ, Fanaroff AA (2013). The preterm lung and airway: past, present, and future. Pediatr Neonatol.

[CR73] Masood A, Yi M, Lau M, Belcastro R, Li J, Kantores C, Pace-Asciak CR, Jankov RP, Tanswell AK (2014). Cyclooxygenase-2 inhibition partially protects against 60% O_2_-mediated lung injury in neonatal rats. Pediatr Pulmonol.

[CR74] McGowan SE, McCoy DM (2015). Fibroblast growth factor signaling in myofibroblasts differs from lipofibroblasts during alveolar septation in mice. Am J Physiol Lung Cell Mol Physiol.

[CR75] McKenna S, Michaelis KA, Agboke F, Liu T, Han K, Yang G, Dennery PA, Wright CJ (2014). Sustained hyperoxia-induced NF-kappaB activation improves survival and preserves lung development in neonatal mice. Am J Physiol Lung Cell Mol Physiol.

[CR76] Miranda LF, Rodrigues CO, Ramachandran S, Torres E, Huang J, Klim J, Hehre D, McNiece I, Hare JM, Suguihara CY, Young KC (2013). Stem cell factor improves lung recovery in rats following neonatal hyperoxia-induced lung injury. Pediatr Res.

[CR77] Mižíková I, Ruiz-Camp J, Steenbock H, Madurga A, Vadász I, Herold S, Mayer K, Seeger W, Brinckmann J, Morty RE (2015). Collagen and elastin cross-linking is altered during aberrant late lung development associated with hyperoxia. Am J Physiol Lung Cell Mol Physiol.

[CR78] Monz D, Tutdibi E, Mildau C, Shen J, Kasoha M, Laschke MW, Roolfs T, Schmiedl A, Tschernig T, Bieback K, Gortner L (2013). Human umbilical cord blood mononuclear cells in a double-hit model of bronchopulmonary dysplasia in neonatal mice. PLoS One.

[CR79] Morrisey EE, Hogan BL (2010). Preparing for the first breath: genetic and cellular mechanisms in lung development. Dev Cell.

[CR80] Morrisey EE, Cardoso WV, Lane RH, Rabinovitch M, Abman SH, Ai X, Albertine KH, Bland RD, Chapman HA, Checkley W, Epstein JA, Kintner CR, Kumar M, Minoo P, Mariani TJ, McDonald DM, Mukouyama YS, Prince LS, Reese J, Rossant J, Shi W, Sun X, Werb Z, Whitsett JA, Gail D, Blaisdell CJ, Lin QS (2013). Molecular determinants of lung development. Ann Am Thorac Soc.

[CR81] Morty RE, Königshoff M, Eickelberg O (2009). Transforming growth factor-beta signaling across ages: from distorted lung development to chronic obstructive pulmonary disease. Proc Am Thorac Soc.

[CR82] Mühlfeld C (2014). Quantitative morphology of the vascularisation of organs: a stereological approach illustrated using the cardiac circulation. Ann Anat.

[CR83] Mühlfeld C, Ochs M (2013). Quantitative microscopy of the lung: a problem-based approach. Part 2. Stereological parameters and study designs in various diseases of the respiratory tract. Am J Physiol Lung Cell Mol Physiol.

[CR84] Nakanishi H, Sugiura T, Streisand JB, Lonning SM, Roberts JD (2007). TGF-beta-neutralizing antibodies improve pulmonary alveologenesis and vasculogenesis in the injured newborn lung. Am J Physiol Lung Cell Mol Physiol.

[CR85] Nardiello C, Morty RE (2016). MicroRNA in late lung development and bronchopulmonary dysplasia: the need to demonstrate causality. Mol Cell Pediatr.

[CR86] Northway WH (1992). Bronchopulmonary dysplasia: twenty-five years later. Pediatrics.

[CR87] Northway WH, Rosan RC, Porter DY (1967). Pulmonary disease following respirator therapy of hyaline-membrane disease. Bronchopulmonary dysplasia. N Engl J Med.

[CR88] Ntokou A, Klein F, Dontireddy D, Becker S, Bellusci S, Richardson WD, Szibor M, Braun T, Morty RE, Seeger W, Voswinckel R, Ahlbrecht K (2015). Characterization of the platelet-derived growth factor receptor-alpha-positive cell lineage during murine late lung development. Am J Physiol Lung Cell Mol Physiol.

[CR89] Null DM, Alvord J, Leavitt W, Wint A, Dahl MJ, Presson AP, Lane RH, DiGeronimo RJ, Yoder BA, Albertine KH (2014). High-frequency nasal ventilation for 21 d maintains gas exchange with lower respiratory pressures and promotes alveolarization in preterm lambs. Pediatr Res.

[CR90] O’Reilly M, Thébaud B (2014). Animal models of bronchopulmonary dysplasia. The term rat models. Am J Physiol Lung Cell Mol Physiol.

[CR91] Ochs M, Mühlfeld C (2013). Quantitative microscopy of the lung: a problem-based approach. Part 1. Basic principles of lung stereology. Am J Physiol Lung Cell Mol Physiol.

[CR92] Olave N, Lal CV, Halloran B, Pandit K, Cuna AC, Faye-Petersen OM, Kelly DR, Nicola T, Benos P, Kaminski N, Ambalavanan N (2015). Regulation of alveolar septation by microRNA-489. Am J Physiol Lung Cell Mol Physiol.

[CR93] Papin JF, Wolf RF, Kosanke SD, Jenkins JD, Moore SN, Anderson MP, Welliver RC (2013). Infant baboons infected with respiratory syncytial virus develop clinical and pathological changes that parallel those of human infants. Am J Physiol Lung Cell Mol Physiol.

[CR94] Park HS, Park JW, Kim HJ, Choi CW, Lee HJ, Kim BI, Chun YS (2013). Sildenafil alleviates bronchopulmonary dysplasia in neonatal rats by activating the hypoxia-inducible factor signaling pathway. Am J Respir Cell Mol Biol.

[CR95] Pham H, Vottier G, Pansiot J, Duong-Quy S, Bollen B, Dalous J, Gallego J, Mercier JC, Dinh-Xuan AT, Bonnin P, Charriaut-Marlangue C, Baud O (2014). Inhaled NO prevents hyperoxia-induced white matter damage in neonatal rats. Exp Neurol.

[CR96] Pieretti AC, Ahmed AM, Roberts JD, Kelleher CM (2014). A novel in vitro model to study alveologenesis. Am J Respir Cell Mol Biol.

[CR97] Poon AW, Ma EX, Vadivel A, Jung S, Khoja Z, Stephens L, Thébaud B, Wintermark P (2016). Impact of bronchopulmonary dysplasia on brain and retina. Biol Open.

[CR98] Popova AP, Bentley JK, Cui TX, Richardson MN, Linn MJ, Lei J, Chen Q, Goldsmith AM, Pryhuber GS, Hershenson MB (2014). Reduced platelet-derived growth factor receptor expression is a primary feature of human bronchopulmonary dysplasia. Am J Physiol Lung Cell Mol Physiol.

[CR99] Quantius J, Schmoldt C, Vazquez-Armendariz AI, Becker C, El Agha E, Wilhelm J, Morty RE, Vadasz I, Mayer K, Gattenloehner S, Fink L, Matrosovich M, Li X, Seeger W, Lohmeyer J, Bellusci S, Herold S (2016). Influenza virus infects epithelial stem/progenitor cells of the distal lung: impact on Fgfr2b-driven epithelial repair. PLoS Pathog.

[CR100] Raffay TM, Locy ML, Hill CL, Jindal NS, Rogers LK, Welty SE, Tipple TE (2013). Neonatal hyperoxic exposure persistently alters lung secretoglobins and annexin A1. Biomed Res Int.

[CR101] Ramachandran S, Suguihara C, Drummond S, Chatzistergos K, Klim J, Torres E, Huang J, Hehre D, Rodrigues CO, McNiece IK, Hare JM, Young KC (2015). Bone marrow-derived c-kit+ cells attenuate neonatal hyperoxia-induced lung injury. Cell Transplant.

[CR102] Ratner V, Sosunov SA, Niatsetskaya ZV, Utkina-Sosunova IV, Ten VS (2013). Mechanical ventilation causes pulmonary mitochondrial dysfunction and delayed alveolarization in neonatal mice. Am J Respir Cell Mol Biol.

[CR103] Rawlins EL (2011). The building blocks of mammalian lung development. Dev Dyn.

[CR104] Richter J, Toelen J, Vanoirbeek J, Kakigano A, Dekoninck P, Verbeken E, Deprest J (2014). Functional assessment of hyperoxia-induced lung injury after preterm birth in the rabbit. Am J Physiol Lung Cell Mol Physiol.

[CR105] Rieger-Fackeldey E, Park MS, Schanbacher BL, Joshi MS, Chicoine LG, Nelin LD, Bauer JA, Welty SE, Smith CV (2014). Lung development alterations in newborn mice after recovery from exposure to sublethal hyperoxia. Am J Pathol.

[CR106] Ruiz-Camp J, Morty RE (2015). Divergent fibroblast growth factor signaling pathways in lung fibroblast subsets: where do we go from here?. Am J Physiol Lung Cell Mol Physiol.

[CR107] Sakurai R, Villarreal P, Husain S, Liu J, Sakurai T, Tou E, Torday JS, Rehan VK (2013). Curcumin protects the developing lung against long-term hyperoxic injury. Am J Physiol Lung Cell Mol Physiol.

[CR108] Salaets T, Richter J, Brady P, Jimenez J, Nagatomo T, Deprest J, Toelen J (2015). Transcriptome analysis of the preterm rabbit lung after seven days of hyperoxic exposure. PLoS One.

[CR109] Schneider JP, Ochs M (2013). Stereology of the lung. Methods Cell Biol.

[CR110] Schneider JP, Ochs M (2014). Alterations of mouse lung tissue dimensions during processing for morphometry: a comparison of methods. Am J Physiol Lung Cell Mol Physiol.

[CR111] Silva DM, Nardiello C, Pozarska A, Morty RE (2015). Recent advances in the mechanisms of lung alveolarization and the pathogenesis of bronchopulmonary dysplasia. Am J Physiol Lung Cell Mol Physiol.

[CR112] Sluiter I, Veenma D, van Loenhout R, Rottier R, de Klein A, Keijzer R, Post M, Tibboel D (2012). Etiological and pathogenic factors in congenital diaphragmatic hernia. Eur J Pediatr Surg.

[CR113] Stoll BJ, Hansen NI, Bell EF, Shankaran S, Laptook AR, Walsh MC, Hale EC, Newman NS, Schibler K, Carlo WA, Kennedy KA, Poindexter BB, Finer NN, Ehrenkranz RA, Duara S, Sanchez PJ, O’Shea TM, Goldberg RN, Van Meurs KP, Faix RG, Phelps DL, Frantz ID, Watterberg KL, Saha S, Das A, Higgins RD, Eunice Kennedy Shriver National Institute of Child H, Human Development Neonatal Research N (2010). Neonatal outcomes of extremely preterm infants from the NICHD Neonatal Research Network. Pediatrics.

[CR114] Sureshbabu A, Syed MA, Boddupalli CS, Dhodapkar MV, Homer RJ, Minoo P, Bhandari V (2015). Conditional overexpression of TGFbeta1 promotes pulmonary inflammation, apoptosis and mortality via TGFbetaR2 in the developing mouse lung. Respir Res.

[CR115] Sutsko RP, Young KC, Ribeiro A, Torres E, Rodriguez M, Hehre D, Devia C, McNiece I, Suguihara C (2013). Long-term reparative effects of mesenchymal stem cell therapy following neonatal hyperoxia-induced lung injury. Pediatr Res.

[CR116] Syed MA, Bhandari V (2013). Hyperoxia exacerbates postnatal inflammation-induced lung injury in neonatal BRP-39 null mutant mice promoting the M1 macrophage phenotype. Mediators Inflamm.

[CR117] Tibboel J, Joza S, Reiss I, de Jongste JC, Post M (2013). Amelioration of hyperoxia-induced lung injury using a sphingolipid-based intervention. Eur Respir J.

[CR118] Tibboel J, Groenman FA, Selvaratnam J, Wang J, Tseu I, Huang Z, Caniggia I, Luo D, van Tuyl M, Ackerley C, de Jongste JC, Tibboel D, Post M (2015). Hypoxia-inducible factor-1 stimulates postnatal lung development but does not prevent O_2_-induced alveolar injury. Am J Respir Cell Mol Biol.

[CR119] Tschanz S, Schneider JP, Knudsen L (2014). Design-based stereology: planning, volumetry and sampling are crucial steps for a successful study. Ann Anat.

[CR120] Tschanz SA, Salm LA, Roth-Kleiner M, Barre SF, Burri PH, Schittny JC (2014). Rat lungs show a biphasic formation of new alveoli during postnatal development. J Appl Physiol.

[CR121] Ueda K, Cho K, Matsuda T, Okajima S, Uchida M, Kobayashi Y, Minakami H, Kobayashi K (2006). A rat model for arrest of alveolarization induced by antenatal endotoxin administration. Pediatr Res.

[CR122] Vadivel A, Alphonse RS, Etches N, van Haaften T, Collins JJ, O’Reilly M, Eaton F, Thébaud B (2014). Hypoxia-inducible factors promote alveolar development and regeneration. Am J Respir Cell Mol Biol.

[CR123] Veenma DC, de Klein A, Tibboel D (2012). Developmental and genetic aspects of congenital diaphragmatic hernia. Pediatr Pulmonol.

[CR124] Wagenaar GT, el Laghmani H, de Visser YP, Sengers RM, Steendijk P, Baelde HJ, Walther FJ (2013). Ambrisentan reduces pulmonary arterial hypertension but does not stimulate alveolar and vascular development in neonatal rats with hyperoxic lung injury. Am J Physiol Lung Cell Mol Physiol.

[CR125] Wagenaar GT, el Laghmani H, Fidder M, Sengers RM, de Visser YP, de Vries L, Rink R, Roks AJ, Folkerts G, Walther FJ (2013). Agonists of MAS oncogene and angiotensin II type 2 receptors attenuate cardiopulmonary disease in rats with neonatal hyperoxia-induced lung injury. Am J Physiol Lung Cell Mol Physiol.

[CR126] Wang H, Jafri A, Martin RJ, Nnanabu J, Farver C, Prakash YS, MacFarlane PM (2014). Severity of neonatal hyperoxia determines structural and functional changes in developing mouse airway. Am J Physiol Lung Cell Mol Physiol.

[CR127] Warburton D, Bellusci S, De Langhe S, Del Moral PM, Fleury V, Mailleux A, Tefft D, Unbekandt M, Wang K, Shi W (2005). Molecular mechanisms of early lung specification and branching morphogenesis. Pediatr Res.

[CR128] Warburton D, El-Hashash A, Carraro G, Tiozzo C, Sala F, Rogers O, De Langhe S, Kemp PJ, Riccardi D, Torday J, Bellusci S, Shi W, Lubkin SR, Jesudason E (2010). Lung organogenesis. Curr Top Dev Biol.

[CR129] Weichelt U, Cay R, Schmitz T, Strauss E, Sifringer M, Buhrer C, Endesfelder S (2013). Prevention of hyperoxia-mediated pulmonary inflammation in neonatal rats by caffeine. Eur Respir J.

[CR130] West JB (2013). Marcello Malpighi and the discovery of the pulmonary capillaries and alveoli. Am J Physiol Lung Cell Mol Physiol.

[CR131] Whitehead GS, Burch LH, Berman KG, Piantadosi CA, Schwartz DA (2006). Genetic basis of murine responses to hyperoxia-induced lung injury. Immunogenetics.

[CR132] Willführ A, Brandenberger C, Piatkowski T, Grothausmann R, Nyengaard JR, Ochs M, Mühlfeld C (2015). Estimation of the number of alveolar capillaries by the Euler number (Euler-Poincare characteristic). Am J Physiol Lung Cell Mol Physiol.

[CR133] Witsch TJ, Niess G, Sakkas E, Likhoshvay T, Becker S, Herold S, Mayer K, Vadász I, Roberts JD, Seeger W, Morty RE (2014). Transglutaminase 2: a new player in bronchopulmonary dysplasia?. Eur Respir J.

[CR134] Witsch TJ, Turowski P, Sakkas E, Niess G, Becker S, Herold S, Mayer K, Vadász I, Roberts JD, Seeger W, Morty RE (2014). Deregulation of the lysyl hydroxylase matrix cross-linking system in experimental and clinical bronchopulmonary dysplasia. Am J Physiol Lung Cell Mol Physiol.

[CR135] Wong PM, Lees AN, Louw J, Lee FY, French N, Gain K, Murray CP, Wilson A, Chambers DC (2008). Emphysema in young adult survivors of moderate-to-severe bronchopulmonary dysplasia. Eur Respir J.

[CR136] Yoder BA, Coalson JJ (2014). Animal models of bronchopulmonary dysplasia. The preterm baboon models. Am J Physiol Lung Cell Mol Physiol.

[CR137] Yoo HS, Chang YS, Kim JK, Ahn SY, Kim ES, Sung DK, Jeon GW, Hwang JH, Shim JW, Park WS (2013). Antenatal betamethasone attenuates intrauterine infection-aggravated hyperoxia-induced lung injury in neonatal rats. Pediatr Res.

[CR138] Zürcher NR, Rodriguez JS, Jenkins SL, Keenan K, Bartlett TQ, McDonald TJ, Nathanielsz PW, Nijland MJ (2010). Performance of juvenile baboons on neuropsychological tests assessing associative learning, motivation and attention. J Neurosci Methods.

